# Mechanical Property Degradation Behavior and Fatigue Life Analysis of Corroded High-Strength Steel Wires

**DOI:** 10.3390/ma19102099

**Published:** 2026-05-16

**Authors:** Guilin Yang, Damin Lu, Lili Jin, Yiqing Zou

**Affiliations:** 1 School of Civil and Architectural Engineering, Guangxi University of Science and Technology, Liuzhou 545006, China; yang5410048@163.com (G.Y.); 100002485@gxust.edu.cn (D.L.); 2 School of Municipal Construction and Transportation, Guangxi Polytechnic of Construction, Nanning 530007, China; litiemei666@163.com; 3 Liuzhou Ovm Machinery Co., Ltd., Liuzhou 530006, China

**Keywords:** electrochemical corrosion, steel wire, mechanical properties, fatigue life, corrosion morphology

## Abstract

Investigating the effects of corrosion on the mechanical and fatigue properties of steel wires is critical for the safety assessment of bridge cable structures.This study focuses on high-strength galvanized steel wires used for bridge cables, with a diameter of 7 mm and a strength grade of 1770 MPa. Specimens with varying mass loss rates *η* were prepared by electrochemical corrosion method, and systematic tensile and fatigue tests were conducted to study the effects of corrosion on the fundamental mechanical properties and fatigue life of the steel wires. The results indicate that the elastic modulus of the steel wires decreases slightly with the increase of *η* but still meets the requirements of relevant standards. In contrast, the yield strength and tensile strength degrade significantly, while ductility is particularly susceptible to corrosion, showing more severe deterioration. When *η* is less than 2.75%, the corroded steel wires still maintain favorable fatigue resistance at a nominal stress amplitude of 360 MPa. Once *η* exceeds this threshold, their fatigue life decreases significantly in a nonlinear manner with increasing *η*. The fatigue life predicted by a finite element model (FEM) reconstructed based on the 3D scanning geometry of corroded steel wires and combined with the Abaqus/fe-safe module shows good agreement with the experimental results, indicating that this approach can provide a valuable reference for the durability assessment of bridge cables.

## 1. Introduction

The service safety of cable-stayed and suspension bridges is highly dependent on the durability of their cable systems [1]. Bridge cables are exposed to complex environmental conditions during long service periods, which makes them prone to corrosion damage and poses a severe threat to structural safety [2,3]. The surface geometric defects induced by corrosion can lead to stress concentrations effects. Under dynamic loads such as traffic, wind, and seismic actions, fatigue cracks are prone to initiate and propagate rapidly, ultimately resulting in premature fracture of individual steel wires or even the entire cable [4,5]. Therefore, investigating the effects of corrosion on the mechanical properties and fatigue behavior of steel wires is a critical issue in the safety assessment of cable structures.

Extensive research has been conducted on the degradation of mechanical properties and fatigue life evaluation of corroded steel wires. Gao et al. investigated the residual strength of steel wires after 18 years of service and identified deep and wide corrosion pits as the dominant factor affecting the of strength steel wire [6]. Xue et al. obtained S-N curves for Zn–Al alloy-coated steel wires via corrosion-fatigue coupling tests and proposed a life prediction model considering the corrosion-fatigue coupling effect [7]. Li et al. examined the mechanical properties of steel wires under stress corrosion, found that stress corrosion accelerates the degradation of mechanical properties, and established a mechanical property degradation model for corroded steel wires [8]. Zhang et al. developed a novel S-N curve incorporating corrosion damage and mean stress for predicting the fatigue life of high-strength steel wires with different corrosion degree [9]. Wang, D.P. investigated the tensile and fatigue properties of steel wires extracted from old suspender rods, estimated residual fatigue life via the Paris formula, and showed good agreement between theoretical predictions and experimental life [10]. Nakamura et al. studied the mechanical properties of steel wires with different corrosion levels and exhibited that the tensile strength remained unchanged, while the elongation, torsional strength, and fatigue strength decreased significantly [11]. Li et al. examined the effects of corrosion duration and stress level on the failure mode, fatigue life, and S-N curves of steel wires, elucidated the crack initiation and propagation mechanisms in corroded steel wires, and proposed a fatigue prediction method considering the corroded surface morphology [12]. Wang et al. proposed an RCSN Weibull model for predicting the fatigue life of corroded steel wires [13]. Tang et al. developed a fatigue life evaluation method based on linear elastic fracture mechanics (LEFM) for predicting the fatigue life of steel wire cables under tension-bending coupling [14]. Tan et al. simulated crack propagation via Abaqus and Franc3D to investigate the variation in stress intensity factors (SIFs) with different crack sizes, stress ratios, and corrosion pit geometries [15]. Ma et al. proposed a subcyclic corrosion fatigue crack growth (CFCG) model to evaluate the damage evolution of bridge suspender steel wires subjected to randomly varying loads [16]. These studies can be categorized into three main research directions: first, obtaining steel wire specimens from decommissioned cables or corrosion tests to conduct mechanical property tests and quantitatively analyze the effects of corrosion on mechanical properties; second, performing fatigue loading tests on corroded steel wires to establish S-N curves and quantify the corrosion-induced reduction in fatigue life; third, combining fracture mechanics theory, the Paris formula, and cumulative damage theory to predict the residual fatigue life under corrosion-fatigue coupling conditions. Although significant progress has been made in various aspects, the findings remain inconsistent due to differences in material types, corrosion morphologies, and experimental methodologies. A unified understanding has yet to be established, and further investigation into the mechanisms of corrosion-induced degradation is warranted.

In the field of numerical simulation, research on refined geometric modeling of corroded bridge cables remains insufficient, with most existing studies relying on idealized geometric defect models such as hemispherical or ellipsoidal corrosion pits for analysis [17,18,19,20]. To a certain extent, such approaches fail to capture the diversity and irregular distribution characteristics of actual corrosion morphologies, and the adoption of simplified models may introduce biases into fatigue durability assessments. In contrast, the method of reconstructing actual corrosion morphology models via three-dimensional (3D) scanning provides a viable solution to address the aforementioned limitations [21,22,23]. Through high-precision point cloud acquisition and surface reconstruction techniques, this method preserves the geometric details and spatial distribution characteristics of surface corrosion morphologies, facilitating the transition from idealized defect models to realistic morphology modeling. Although this method has been widely applied in fields such as mechanical engineering and aerospace, its application in the study of bridge cable corrosion remains limited.

Therefore, this study focuses on high-strength galvanized steel wires for bridge cables with a diameter of 7 mm and a strength grade of 1770 MPa. Specimens with varying *η* were prepared via electrochemical corrosion, tensile and fatigue tests were further conducted to investigate the influence pattern of corrosion on the mechanical properties and fatigue life of the wires. Combined with fatigue fracture analysis, the fatigue failure mechanism of corroded wires was further elucidated. Based on reverse modeling via 3D scanning, fatigue life prediction analysis was performed using Abaqus/fe-safe to validate the fatigue life prediction method considering actual corrosion morphologies, providing a scientific basis and technical reference for the full-lifecycle safety assessment and maintenance decision-making of bridge cables.

## 2. Materials and Methods

### 2.1. Test Specimens

**Figure 1:**
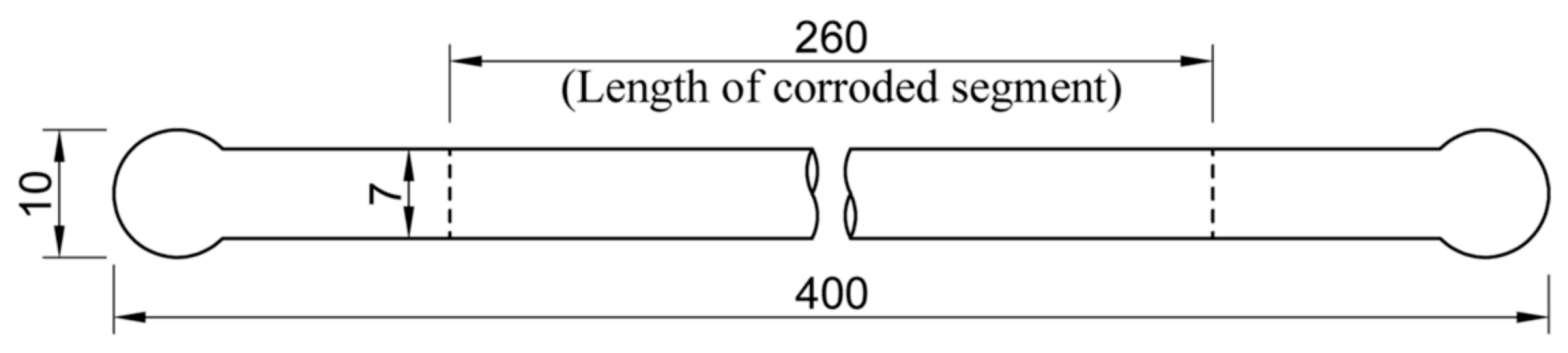
Schematic diagram of the geometric dimensions of steel wire specimens (mm).

The tests employed high-strength galvanized steel wires with a nominal diameter of 7 mm and a specified tensile strength grade of 1770 MPa. All specimens underwent cold heading treatment at both ends to facilitate secure gripping during mechanical testing. The measured baseline mechanical properties of the uncorroded wires are summarized in Table 1, and the specimen geometry is shown in Figure 1. The middle 260 mm segment of the specimen is designated as the target corrosion region.

### 2.2. Electrochemical Corrosion Test

**Figure 2:**
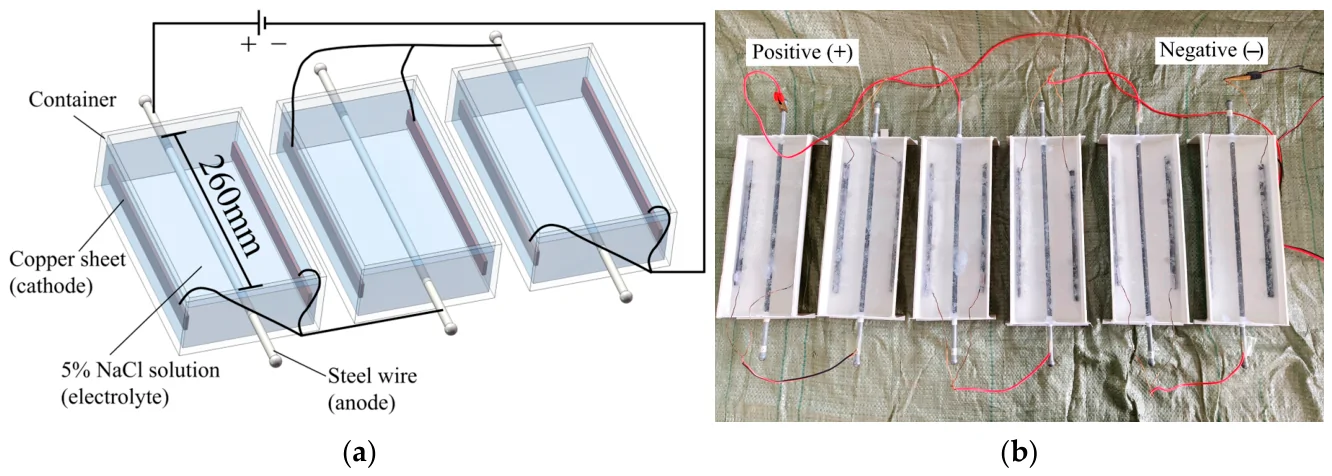
Electrochemical corrosion test setup and process: (**a**) Schematic diagram of the electrochemical corrosion device; (**b**) Corrosion process.

Corroded steel wire specimens were prepared through an electrochemical accelerated corrosion method, with different mass loss rates *η* obtained by controlling the corrosion duration. A total of 42 specimens were divided into seven groups (designated A to G), each comprising six specimens. Group A served as the uncorroded control, while Groups B to G were subjected to electrochemical corrosion for 12 h, 24 h, 48 h, 72 h, 96 h, and 120 h, respectively. Prior to corrosion, the initial mass *m*_0_ of each specimen was measured using an electronic balance with an accuracy of 0.01 g. The electrolyte was a 5 wt% NaCl solution, and a copper sheet was employed as the auxiliary electrode. The current density was maintained at 2370 µA/cm^2^, corresponding to a current of 135 mA. To ensure consistent *η* values among specimens within the same group, the steel wires were connected in series throughout the test, as illustrated in Figure 2. Upon completion of corrosion, the specimens were removed and subjected to electrolytic rust removal in accordance with the standard Corrosion of Metals and Alloys—Removal of Corrosion Products from Corrosion Test Specimens [24], after which the residual mass *m*_1_ was determined.

### 2.3. Test Tensile and Fatigue Test

**Figure 3:**
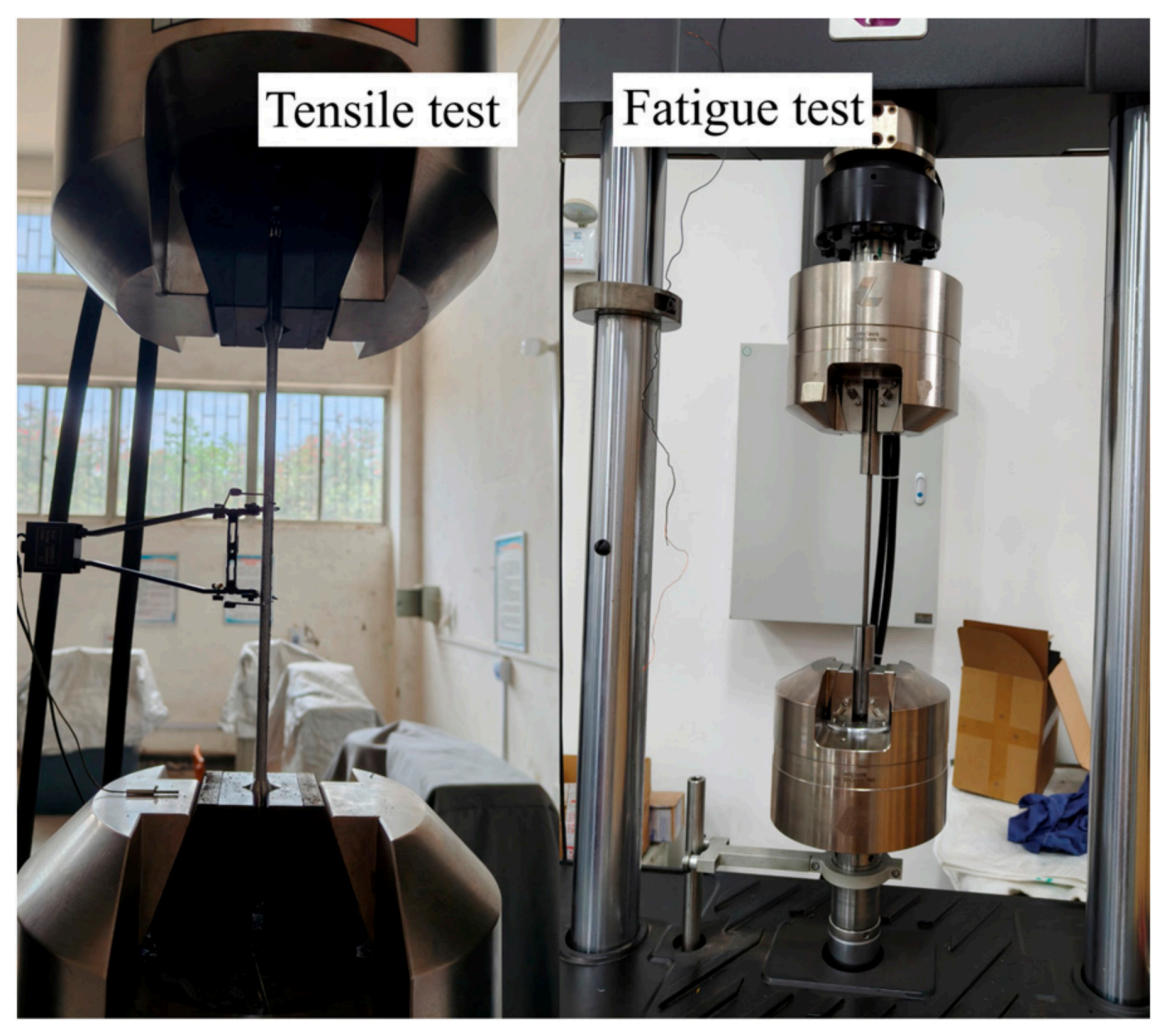
Tensile and fatigue tests.

Tensile tests were conducted on an electronic universal material testing machine under displacement control at a loading rate of 1 mm/min, with strain monitored using a 50 mm extensometer. Fatigue tests were performed on an electrohydraulic servo universal testing machine under load control, employing a sinusoidal waveform at a frequency of 10 Hz. The test setup is illustrated in Figure 3. Nominal stress was calculated based on the original cross-sectional area of the uncorroded steel wires to determine the applied load amplitudes. In accordance with the standard Hot-dip Galvanized or Zinc–Aluminum Alloy Coated Steel Wires for Bridge Cables (GB/T 17101-2019) [25], the maximum stress *σ_max_* was set to 45% of the nominal tensile strength (797 MPa). Accordingly, the stress amplitude *Δσ* and minimum stress *σ_min_* were set to 360 MPa and 437 MPa, respectively, based on the nominal cross-sectional area of the steel wires. The corresponding peak and minimum tensile loads were calculated as 30.672 kN and 16.818 kN. The test was automatically terminated when the steel wire fractured or the cyclic number reached 2 million.

## 3. Test Results and Discussion

### 3.1. Electrochemical Corrosion Test Results

#### 3.1.1. Morphology Evolution of Corroded Steel Wires

**Figure 4:**
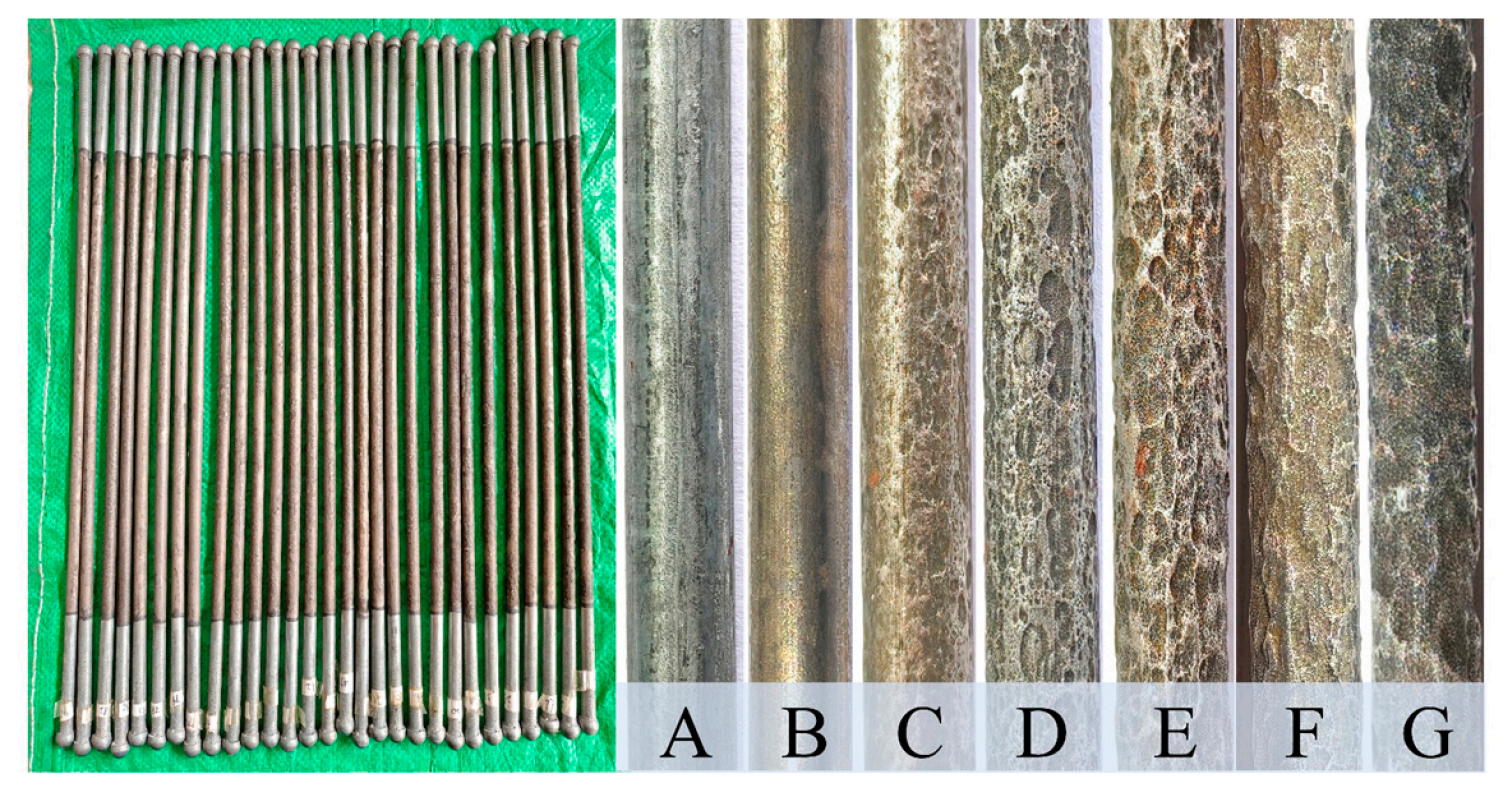
Changes in surface morphology of corroded steel wires. (A–G) Representative magnified surface morphologies of steel wires from Groups A to G, respectively.

Figure 4 illustrates the surface morphologies of corroded steel wires, along with representative local magnifications for each group. Group A corresponds to the uncorroded steel wires. For Group B, the galvanized coating was not fully consumed, and only localized micro-pits were observed. With progressive corrosion, these surface pits gradually coalesced to form deep, wide corrosion cavities, ultimately producing extensive contiguous corroded regions.

#### 3.1.2. Mass Loss Rate Statistics

**Figure 5:**
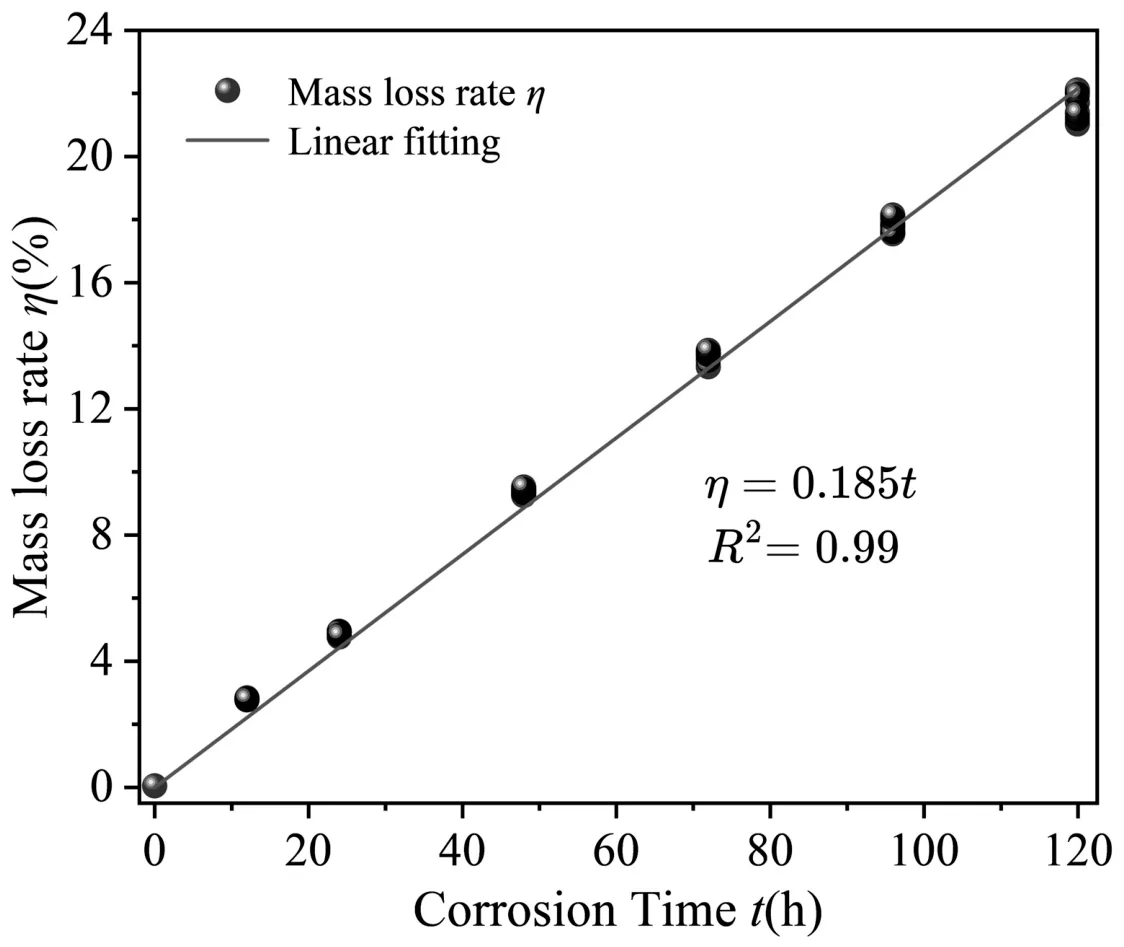
Variation of *η* with corrosion time.

In this study, the mass loss rate *η* was adopted to quantify the degree of corrosion of the steel wires. Following the corrosion tests, *η* for each specimen was calculated using Equation (1), with the results summarized in Table 2.

(1) $$\eta =\frac{m_{0}-m_{1}}{m}\text{×}100\%,$$

where *m*_0_ is the total mass of the full-length uncorroded specimen, *m*_1_ represent the residual mass of the full-length corroded specimen after the removal of corrosion products, and *m* denotes the mass of an uncorroded steel wire of 260 mm in length. The mean value of *η* for the six specimens in each group was adopted as the average for that taken group. Figure 5 further illustrates the variation tendency of *η* with corrosion time, which was fitted by a linear function. The results reveal a significant linear correlation between *η* of the steel wire and the corrosion time.

### 3.2. Reconstruction of Corroded Wire Models

**Figure 6:**
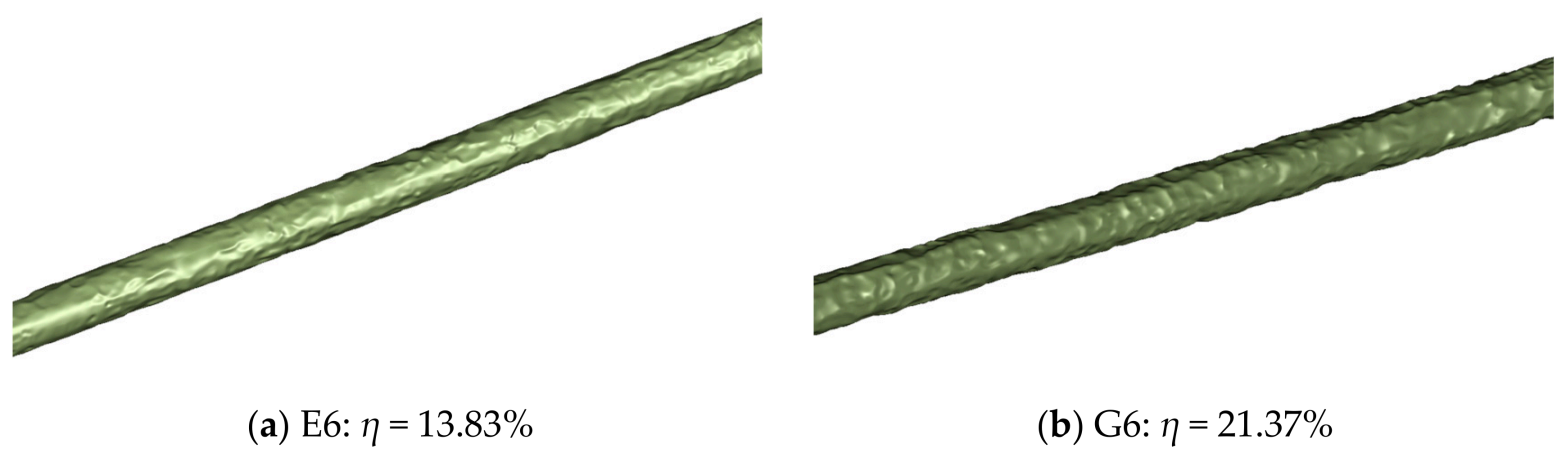
3D Model of Corrosion Steel Wire.

To construct finite element models incorporating realistic corrosion surface topographies, one representative specimen from each group was selected for high-precision three-dimensional (3D) laser scanning to acquire surface point cloud data. The resulting datasets were subsequently processed using Geomagic Wrap for denoising, surface wrapping, and reconstruction, yielding geometric models that faithfully capture detailed features such as pits and corrosion grooves, as illustrated in Figure 6.

**Figure 7:**
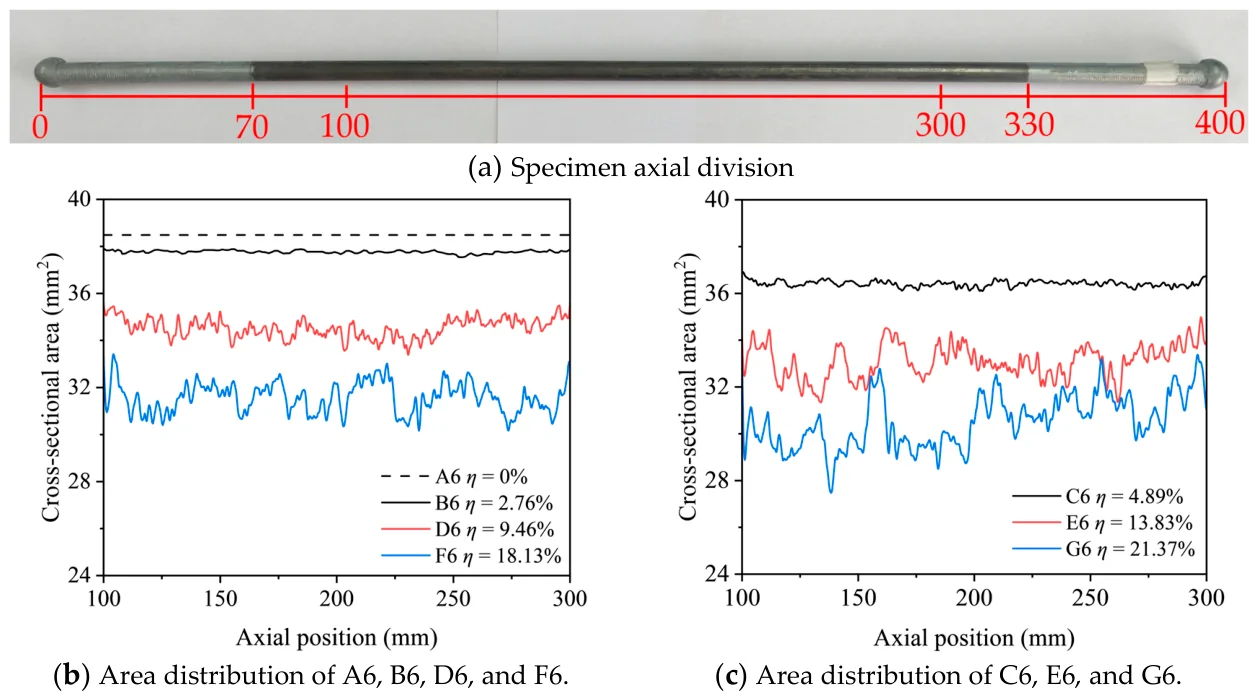
Axial distribution of cross-sectional area.

To preclude potential artifacts arising from inhomogeneous electric field distributions near the wire ends during corrosion testing, a 200 mm central segment of each specimen was selected as the representative gauge region. Based on the 3D model, cross-sectional areas were extracted along the longitudinal axis at 0.2 mm intervals, with as shown in Figure 7. It is evident that the cross-sectional area of the steel wire decreases significantly with increasing *η*, while the fluctuation of cross-sectional area becomes more pronounced along the axial direction of the specimen. At low *η* values (specimens B6 and C6), cross-sectional areas remain relatively high and exhibit only modest variation, indicating that corrosion proceeds predominantly through the nucleation and independent growth of isolated pits. For *η* exceeding 9.46%, the amplitude of area fluctuations intensifies substantially, signifying the coalescence of individual pits into broader, deeper cavities and a morphological transition from quasi-uniform to distinctly non-uniform corrosion.

Table 3 lists the minimum cross-sectional area *S_min_* and average cross-sectional area *S_avg_* of steel wires extracted based on the reconstructed 3D model. With the increase of *η*, both *S_min_* and *S_avg_* show a downward trend, and the difference between the two continues to increase as corrosion intensifies. For specimens with *η* (B6 and C6), the difference between *S_min_* and *S_avg_* is approximately within the range of 0.2–0.3 mm^2^, indicating that corrosion at this stage is dominated by uniform thinning and the overall uniformity of the steel wire cross-section is good; By comparison, for the G6 steel wire with a high *η* of 21.37%, the difference reaches 3.071 mm^2^, and the cross-sectional non-uniformity increases significantly.

### 3.3. Tensile Test Results and Discussion

#### 3.3.1. Stress–Strain Curves of Steel Wires

**Figure 8:**
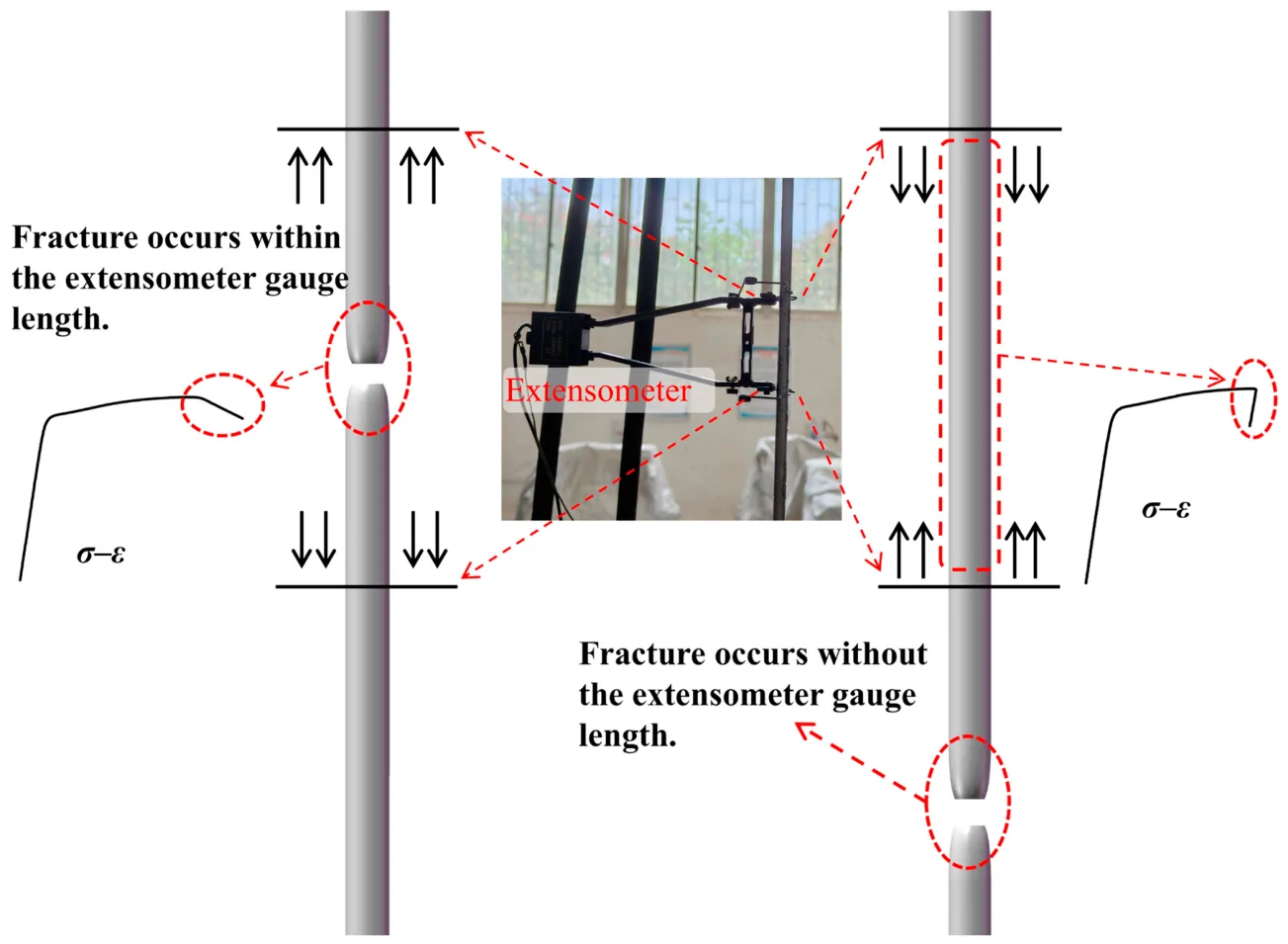
Schematic diagram of steel wires fracturing at different positions.

The average residual cross-sectional area *S_η_* of the corroded segment of each steel wire was calculated using Equation (2), based on which the stress-strain (*σ*–*ε*) curves were derived.

(2) $$S_{\eta }=S_{0}\;(1-\eta ),$$

where *S_η_* denotes the average residual cross-sectional area, and *S*_0_ is the initial cross-sectional area. It should be noted that during tensile testing, some steel wires fractured without extensometer gauge length (as shown in Figure 8), and thus the descending portions of the corresponding *σ*–*ε* curves exhibit a retraction behavior.

**Figure 9:**
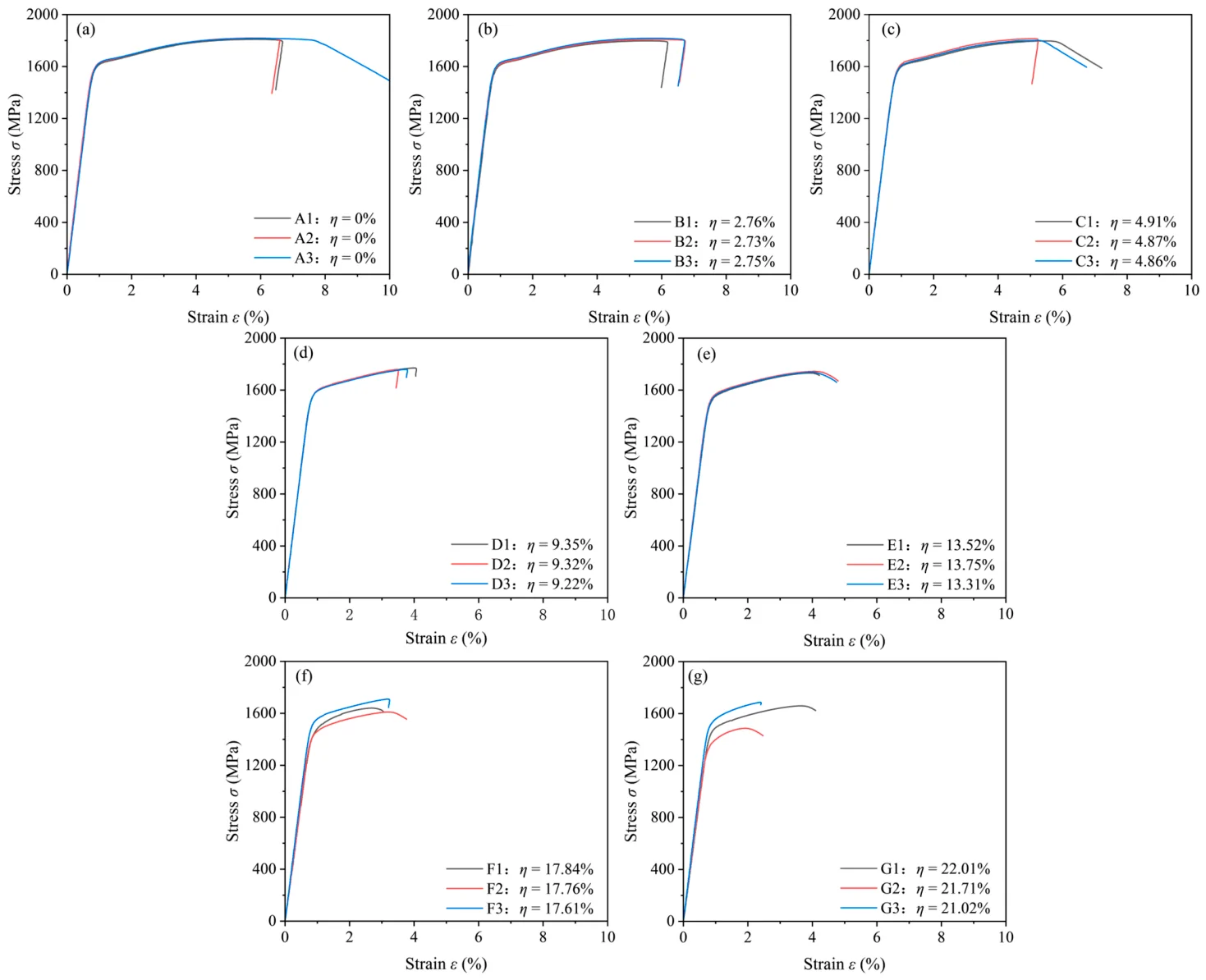
*σ*–*ε* curves of steel wire under different *η*. (**a**): Groups A; (**b**): Groups B; (**c**): Groups C; (**d**): Groups D; (**e**): Groups E; (**f**): Groups F; (**g**): Groups G.

Figure 9 illustrates the *σ*–*ε* curves of steel wires with different *η*. It can be observed that the slopes of the elastic segments of the curves for Groups A–E are generally consistent, while those for Groups F and G exhibit slight fluctuations. With increasing *η*, the yield strength gradually decreases, and the degree of strain hardening diminishes, indicating that corrosion simultaneously impairs both the plastic deformation capacity and load-bearing capacity of the steel wires. The peak stress gradually decreases with increasing *η*, accompanied by a continuous reduction in ultimate strain and a significant decrease in fracture strain, reflecting an embrittlement trend in the corroded steel wires.

#### 3.3.2. Changes in Mechanical Performance Indicators

**Figure 10:**
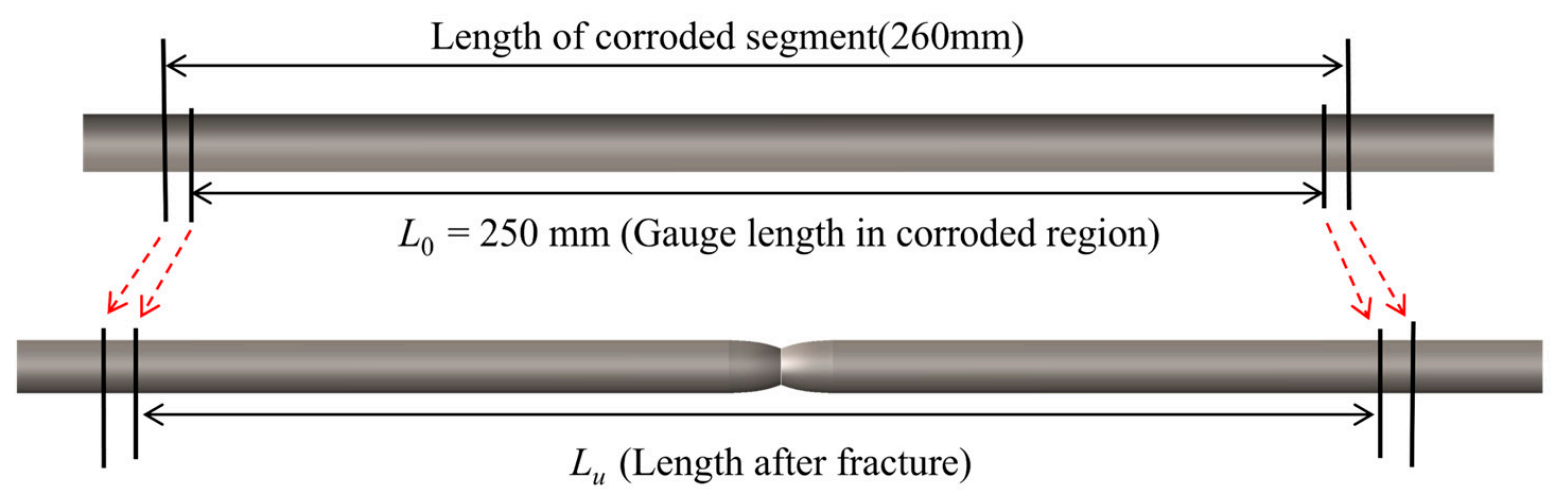
Schematic diagram for calculation of percentage elongation after fracture *A*.

Based on the aforementioned *σ*–*ε* curves, the mechanical property indicators of each corroded steel wire were extracted. The elastic modulus *E* was determined as the slope of the linear fit to the elastic segment of the *σ*–*ε* curve. The yield strength *σ_y_* was defined as the stress corresponding to 0.2% plastic strain [26], and the tensile strength *σ_u_* was taken as the maximum stress on the *σ*–*ε* curve. The ductility of the steel wires was characterized by the percentage elongation after fracture *A*, which was calculated using Equation (3), with the measurement schematic illustrated in Figure 10.

(3) $$A=\frac{L_{u}-{\;L}_{0}}{L_{0}}\text{×}100\%,$$

where *L*_0_ denotes the standard gauge length for elongation measurement (set to 250 mm), and *L_u_* is the combined length of the two fractured segments after testing.

**Figure 11:**
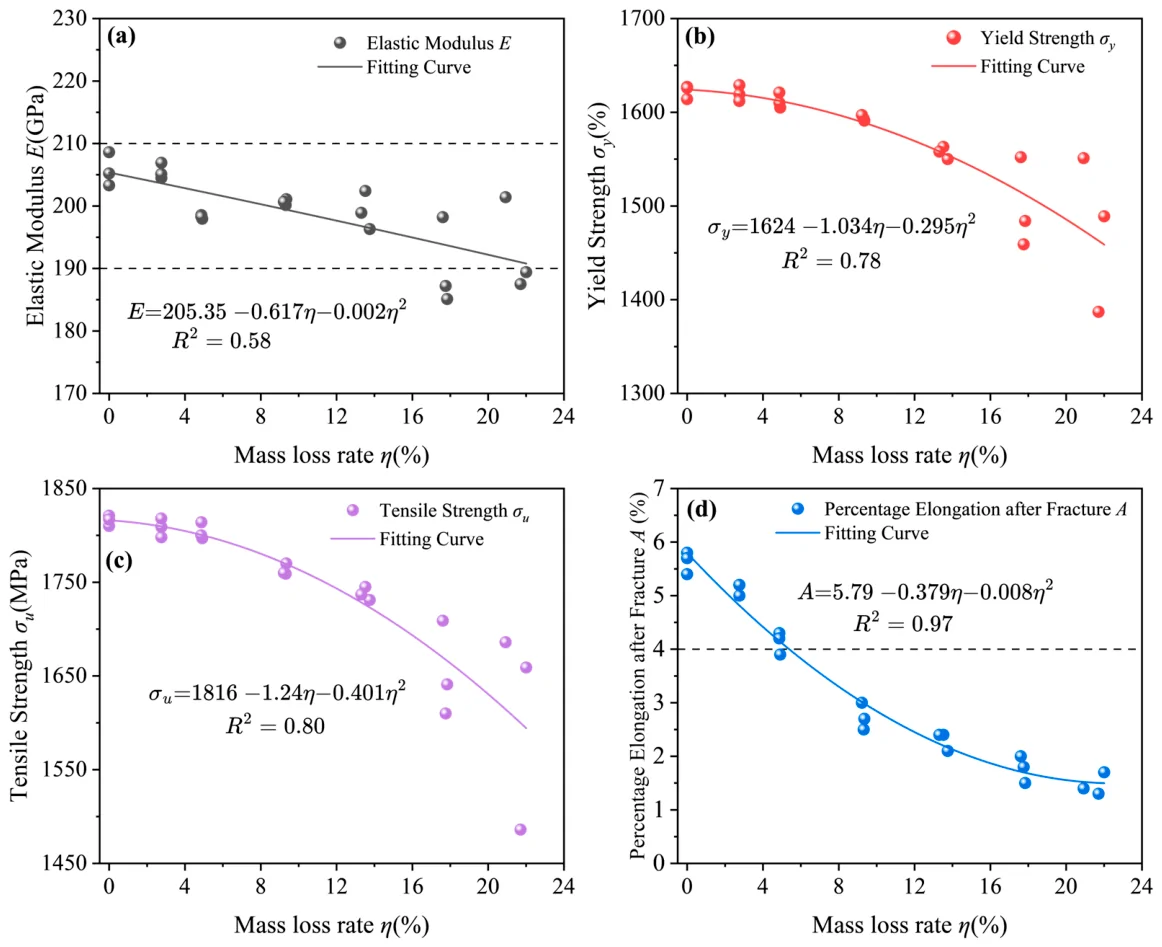
Variation of mechanical property indicators with *η*. (**a**–**d**) Variations of E, *σ_y_*, *σ_u_*, and *A* with *η*, respectively; dashed lines denote standard-specified limits.

Figure 11 illustrates the scatter plots of the mechanical property indicators as a function of *η*, along with fitted curves for reference. As illustrated in Figure 11a, the elastic modulus of the steel wires decreases slightly with increasing *η*. Most of the measured elastic modulus values fall within the range of 200 ± 10 GPa, which still meets the standard requirements [25].

Figure 11b,c indicate that both the yield strength and the tensile strength show a declining trend as *η* increases. This trend is characterized by a slower initial decrease followed by a more rapid decline. When the average *η* is less than 9.36%, the magnitude of strength reduction is relatively small. When the average *η* reached 21.54% (Group G), the average yield strength decreased to 1476 MPa, a reduction of 9.0%, while the average tensile strength dropped to 1610 MPa, a reduction of 11.3%. As the *η* increased, the data points for both yield strength and tensile strength became increasingly scattered, indicating that the non-uniformity of corrosion exerts a growing influence on the mechanical properties of the steel wires. In some individual steel wires, severe localized corrosion led to performance degradation at a rate significantly exceeding the average.

Figure 11d demonstrates that the ductility of the steel wires is highly sensitive to corrosion. Even at a low corrosion degree with an *η* of merely 2.75% (Group B), the mean elongation after fracture decreased from 5.6% (for uncorroded steel wires) to 5.1%, a reduction of approximately 9%. When the average *η* increases to 4.84% (Group C), the average elongation dropped to 4.1%, approaching the minimum standard requirement of 4%. For the corroded steel wires with an *η* of 21.54% (Group G), the average elongation was merely 1.5%, corresponding to a substantial reduction of 73.2%, indicating a pronounced embrittlement tendency. Overall, the percentage elongation after fracture of the steel wires exhibits a decreasing trend characterized by a rapid initial decline followed by a slower decrease, which contrasts with the degradation pattern observed for strength.

### 3.4. Fatigue Test Results and Discussion

**Figure 12:**
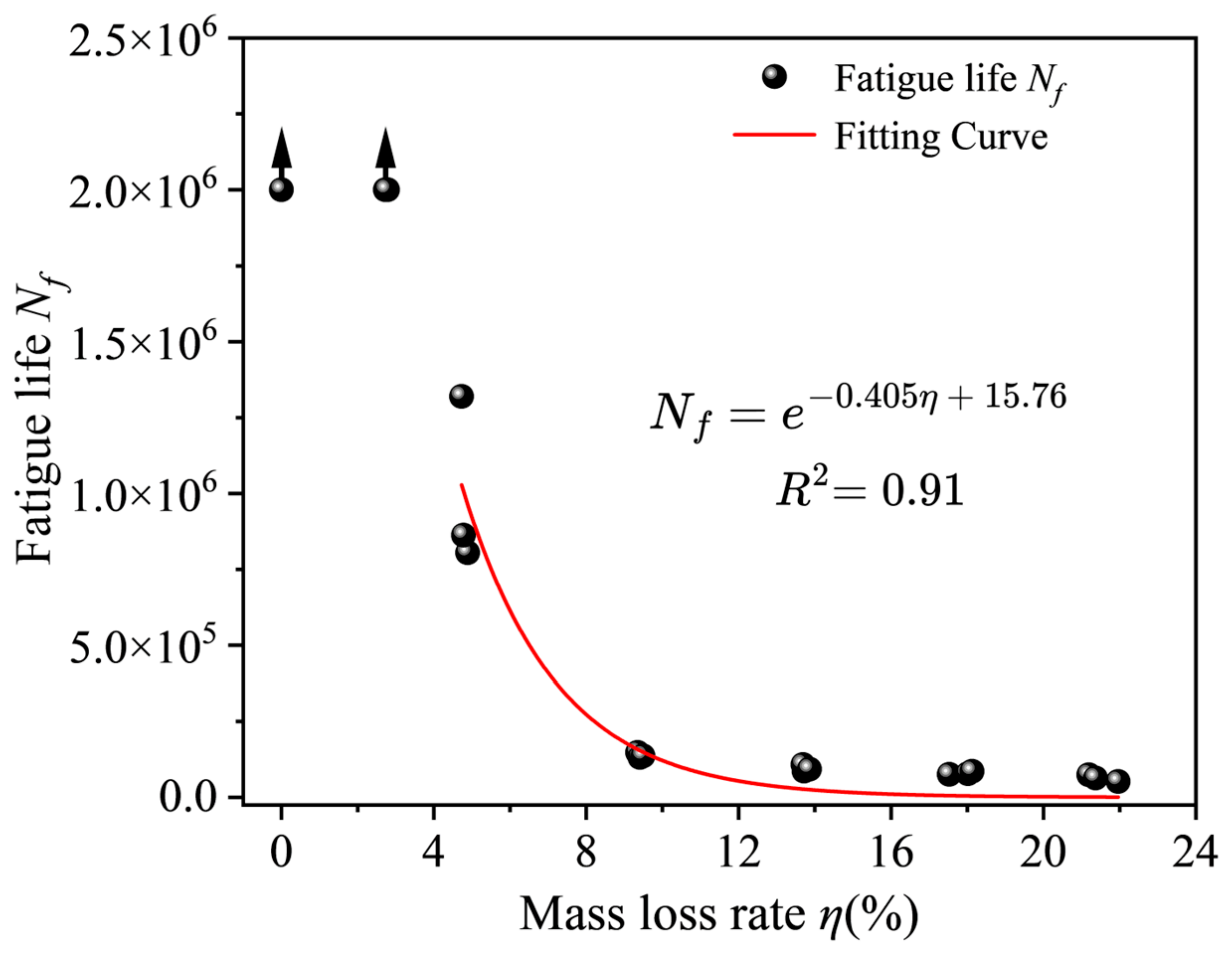
Variation of fatigue life with *η*. “↑” indicates *N_f_* > 2,000,000.

Table 4 summarizes the fatigue life *N_f_* of each corroded steel wire specimen tested in this study, along with the corresponding average values. Figure 12 illustrates the variation of fatigue life with *η* using scatter plots and a fitted curve. The results indicate that both the uncorroded steel wires (Group A) and those with *η* of 2.75% (Group B) endured 2 million loading cycles without fracture, demonstrating that these specimens retained good fatigue resistance. When *η* increased to 4.84% (Group C), the fatigue life of the steel wires declined sharply, with the average fatigue life decreasing to approximately 1,000,000 cycles, representing a reduction of more than 50%. At *η* of 21.54% (Group G), the average fatigue life was only 63,304 cycles, which is less than 3.2% of that of the uncorroded steel wires.

To more intuitively illustrate the trend of fatigue life variation, a nonlinear fit was performed on the data. Since the steel wire specimens in Groups A and B did not experience fatigue fracture after 2 million cycles, their actual fatigue life remains unknown. Therefore, these two groups were excluded from the fitting, and the resulting equation is as follows:

(4) $$N_{f}\;=\;{e\;}^{-0.405\eta \;+\;15.76},$$

This equation effectively quantifies the relationship between the two variables, revealing a pronounced exponential decay trend in the fatigue life of the steel wires as *η* increases.

### 3.5. Fracture Surface Morphology Analysis

**Figure 13:**
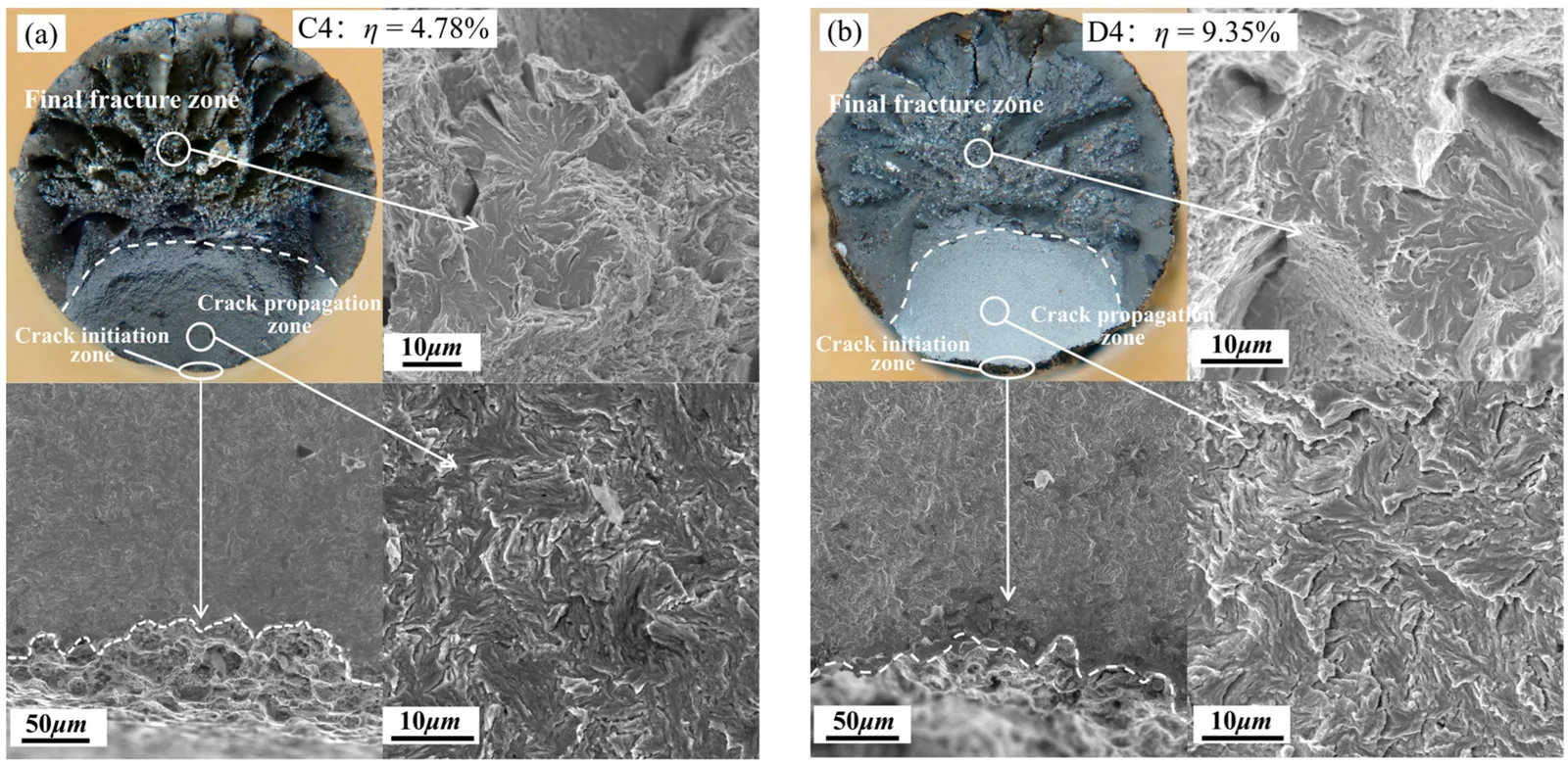

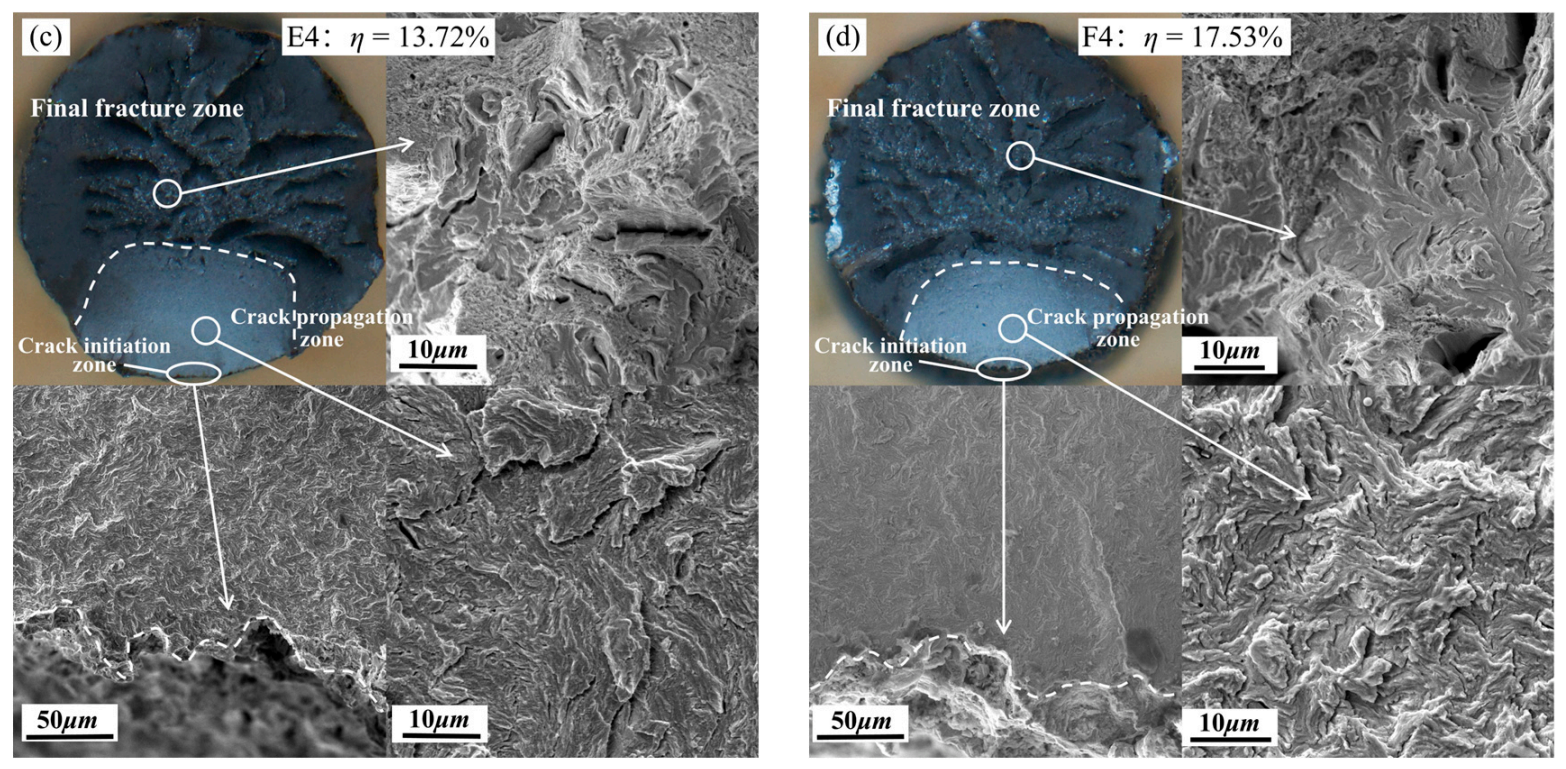
Comparison of macroscopic and microscopic morphologies of fatigue fractures in steel wires with different *η*. (**a**) C4: *N_f_* = 862,894; (**b**) D4: *N_f_* = 148,137; (**c**) E4: *N_f_* = 86,137; (**d**) F4: *N_f_* = 77,754.

Figure 13 presents the macroscopic morphologies and corresponding microscopic SEM images of fatigue fracture surfaces of steel wires subjected to varying *η*. Macroscopically, all fracture surfaces exhibit the characteristic tripartite zonation comprising the crack initiation zone, the crack propagation zone, and the final fracture zone. However, the relative area fractions of these zones vary distinctly with corrosion severity. Specifically, the area fraction of the propagation zone decreases sequentially from approximately 27.5% for specimen C4 to 20.2% for F4 (C4: 27.5%, D4: 24.6%, E4: 22.8%, F4: 20.2%). This phenomenon implies that the fatigue crack propagation life decreases with the increase of *η*.

High-magnification micrographs of the fatigue fracture surface show that corrosion pits on the surface of steel wires with different *η* are all composed of micro-pits of varying sizes and irregular shapes, and fatigue cracks predominantly initiate at the bottom of these corrosion pits. The crack propagation zone exhibits transgranular ductile fracture characteristics, where irregular fatigue striations also can be clearly observed, the spacing of fatigue striations characterizes the crack growth distance driven by stress per cycle. In the final fracture zone, all specimens exhibit a mixed fracture mode of dimples and quasi-cleavage, along with observable microvoids and secondary cracks.

Based on the above analysis, the mechanism by which corrosion accelerates fatigue failure can be summarized in two stages. In the crack initiation stage, surface corrosion pits act as stress concentration sites, accelerating the rate of cyclic plastic accumulation in the local region at the pit base, thereby inducing microcrack formation after fewer loading cycles. In the crack propagation stage, corrosion reduces the effective load-bearing cross-sectional area of the steel wire. As a result, under the same applied load, the actual stress level experienced by the steel wire increases significantly—that is, the driving force for crack propagation is greatly enhanced—thereby accelerating the crack propagation rate. Therefore, pre-corrosion does not change the essential properties of steel wire materials. Corrosion significantly reduces the fatigue life of high-strength galvanized steel wire, which results from the combined effects of geometric morphology deterioration and mechanical loading effects induced by corrosion.

## 4. Numerical Simulation

### 4.1. Finite Element Model and Fatigue Analysis Setup

**Figure 14:**
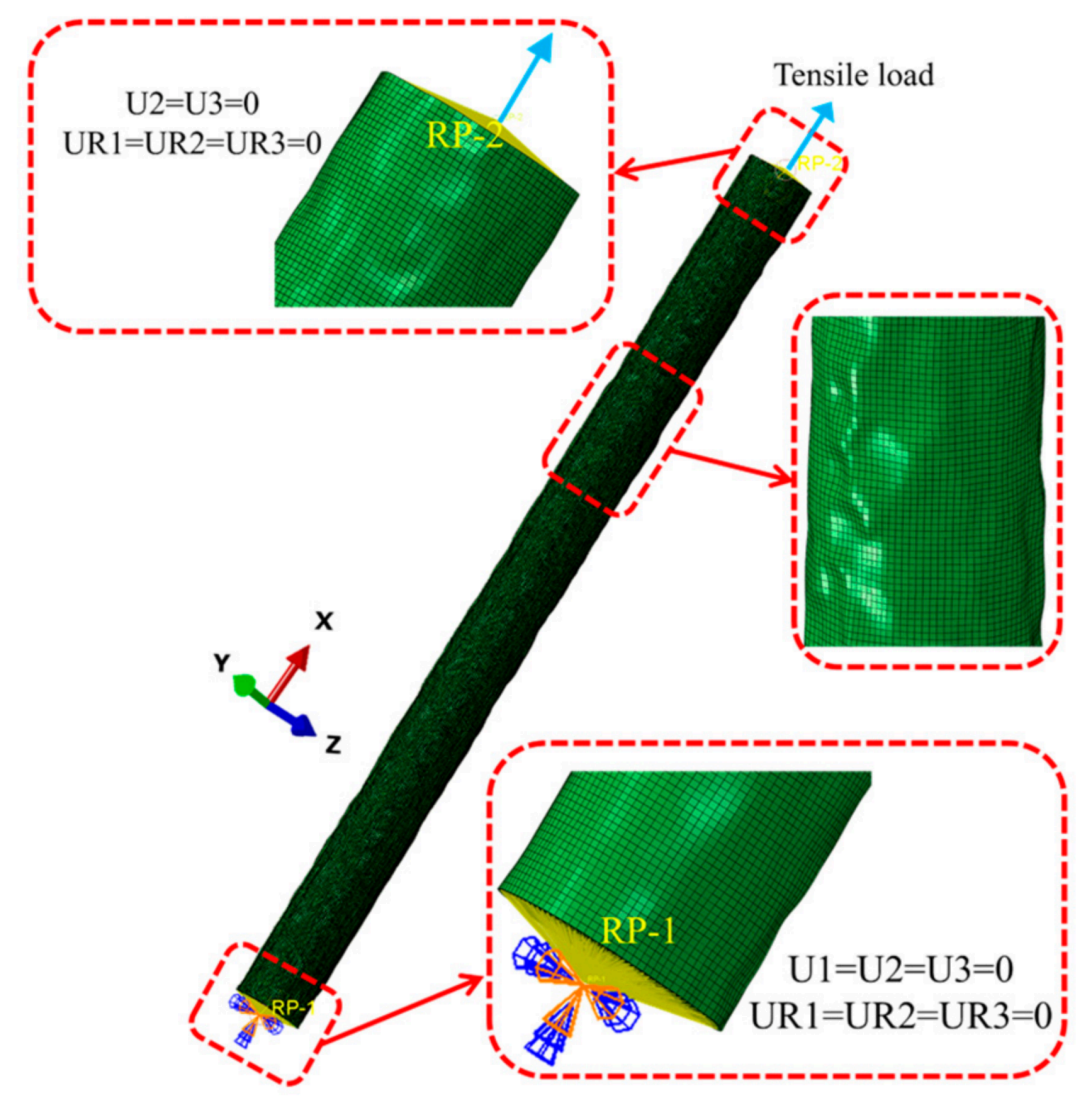
Mesh and boundary conditions of the steel wire finite element model.

**Figure 15:**
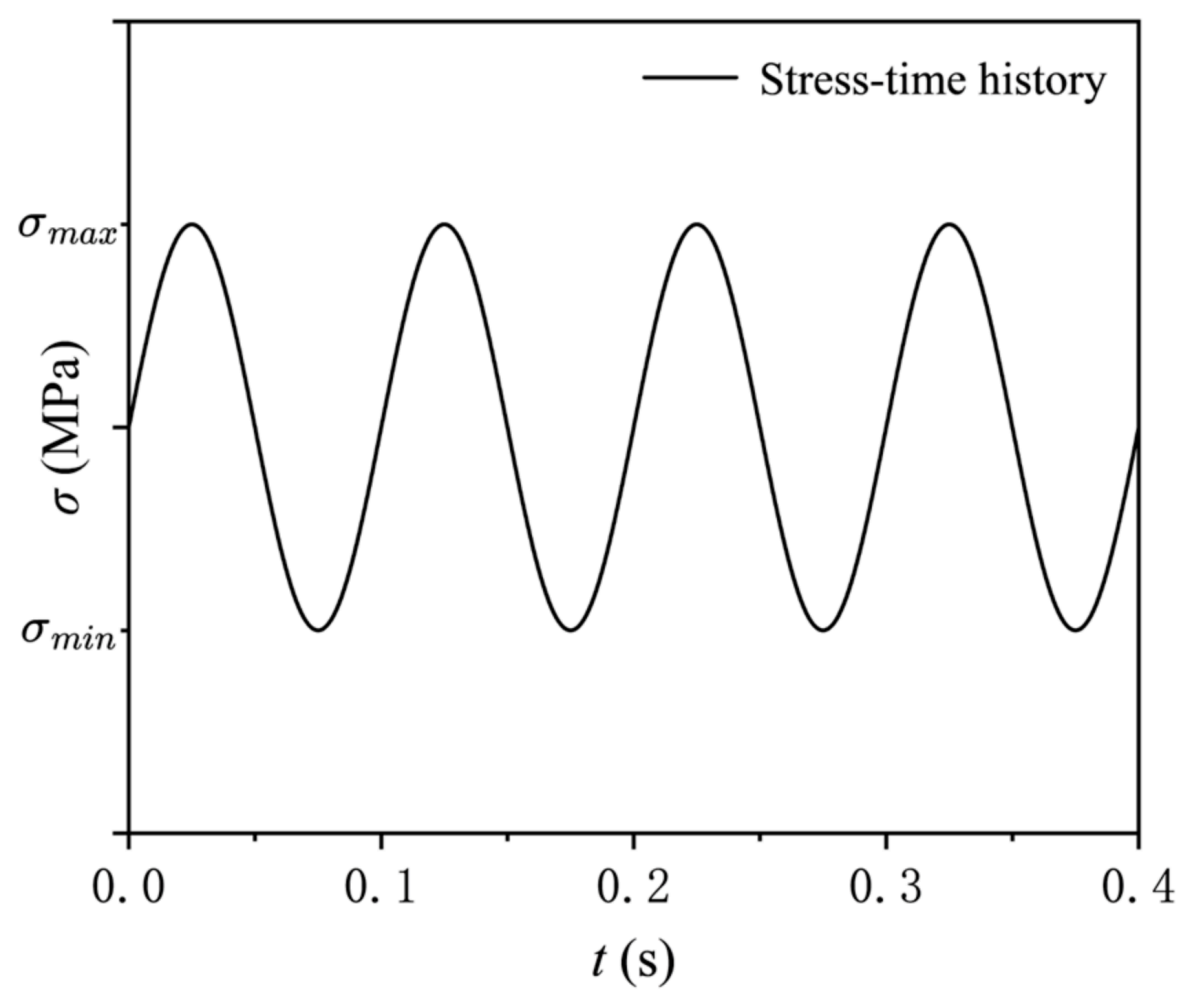
Stress-time history curve.

The steel wire model with actual corrosion surfaces obtained previously was imported into Abaqus for stress calculations. Based on the analysis in Section 3.5, pre-corrosion does not alter the intrinsic material properties of the steel wires. Thus, the material parameters for the FEM were defined based on the uncorroded steel wires, with the measured tensile test data as input and Poisson’s ratio set to 0.3. The geometry was discretized using eight-node linear brick elements with reduced integration (C3D8R), with a global mesh size of approximately 0.2 mm. For the boundary conditions, one end of the model was fixed, and an axial tensile load was applied to the opposite end via coupling constraints with a reference point (RP). To simulate the fatigue test loading process described in Section 2.3, two analysis steps were established. In Step-1, an axial tensile force of 16.818 kN was applied, followed by Step-2, in which the load was increased to 30.672 kN. The FEM setup is illustrated in Figure 14. The computed stress results were then imported into the fe-safe software 2022, where the maximum stresses from the two analysis steps were extracted to serve as *σ_min_* (the stress at 16.818 kN) and *σ_max_* (the stress at 30.672 kN) in the stress time history curve, as shown in Figure 15.

In fe-safe, the S-N curve-based fatigue algorithm was selected, and the material parameters were defined according to the experimental data. The S-N curve used in this study was obtained from Reference [27]. Prior to this study, multiple fatigue validation tests were conducted on the same batch of steel wires under the same stress amplitude as that in the referenced study, with the test results consistent with the S-N curve presented therein.

### 4.2. Numerical Simulation Results and Discussion

**Figure 16:**
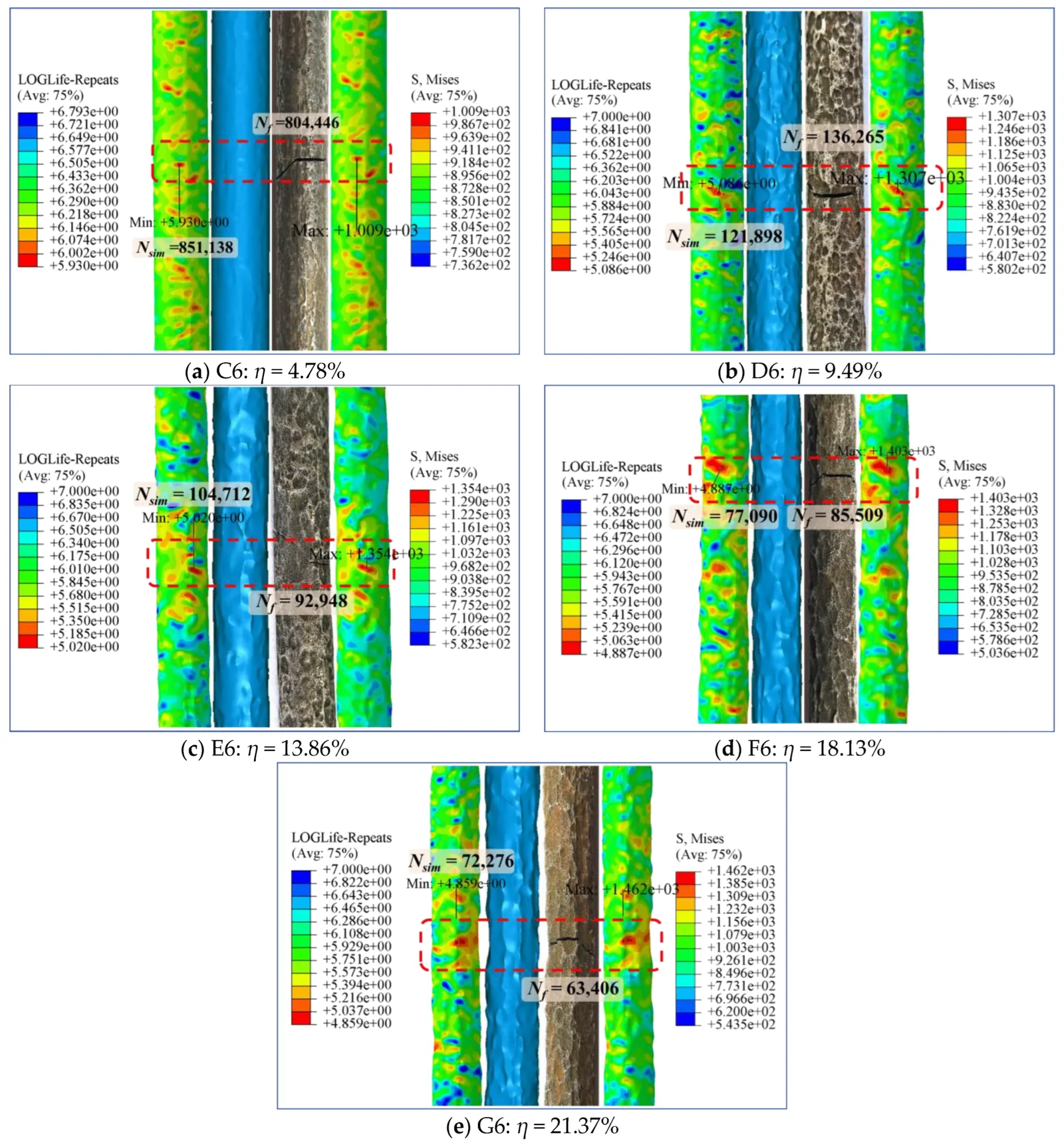
Comparison of numerical simulation and experimental results for steel wires under different *η*.

Figure 16 illustrates the numerical simulation results for steel wires C6–G6. For each specimen, the figure shows, from left to right, the fatigue life distribution contour plot, the scan-reconstructed 3D model, the corresponding experimental fatigue fracture photograph, and the von Mises stress *σ_vm_* distribution contour plot under the maximum fatigue load (30.672 kN). 

The nominal stress calculated based on the uncorroded steel wire with a diameter of 7 mm is 797MPa. Specifically, the maximum Mises stress of steel wires with *η* of 4.89%, 9.49%, 13.86%, 18.13%, and 21.37% are 1009 MPa, 1307 MPa, 1354 MPa, 1403 MPa, and 1462 MPa, respectively. Compared with the nominal stress level of uncorroded steel wires, the growth amplitudes of the maximum Mises stress are 26.6%, 64.0%, 69.9%, 76.0%, and 83.4%, respectively, indicating that the corrosion-induced stress concentration effect increases becomes increasingly severe with the increasing *η*. Further, the region of the minimum simulated fatigue life coincides with the location of both the experimental fatigue failure and maximum Mises stress, as highlighted by red box. This indicates that the proposed method can effectively predict the failure locations of corroded steel wires and predict fatigue life.

Meanwhile, distinct stage-wise characteristics can be observed in the dominant fatigue failure mechanism. At *η* = 4.78%, the red regions in the contour plots appear as discrete points, indicating that the fatigue failure mechanism of the wire is primarily controlled by the stress concentration at discrete corrosion pits. When *η* = 9.49%, the high-stress regions gradually become localized and exhibit a band-like distribution, suggesting that the stress fields of adjacent pits interact and the fatigue failure mechanism has transitioned to a multi-pit coupling effect. For *η* ≥ 13.86%, the high-stress regions are distributed in transverse bands, and the area of these bands continues to expand, demonstrating that the fatigue failure of the wire is dominated by the remaining effective load-bearing cross-sectional area, while the influence of pit geometry is weakened.

In addition, the fatigue life distribution contour plots are highly consistent with the von Mises stress distribution.

**Figure 17:**
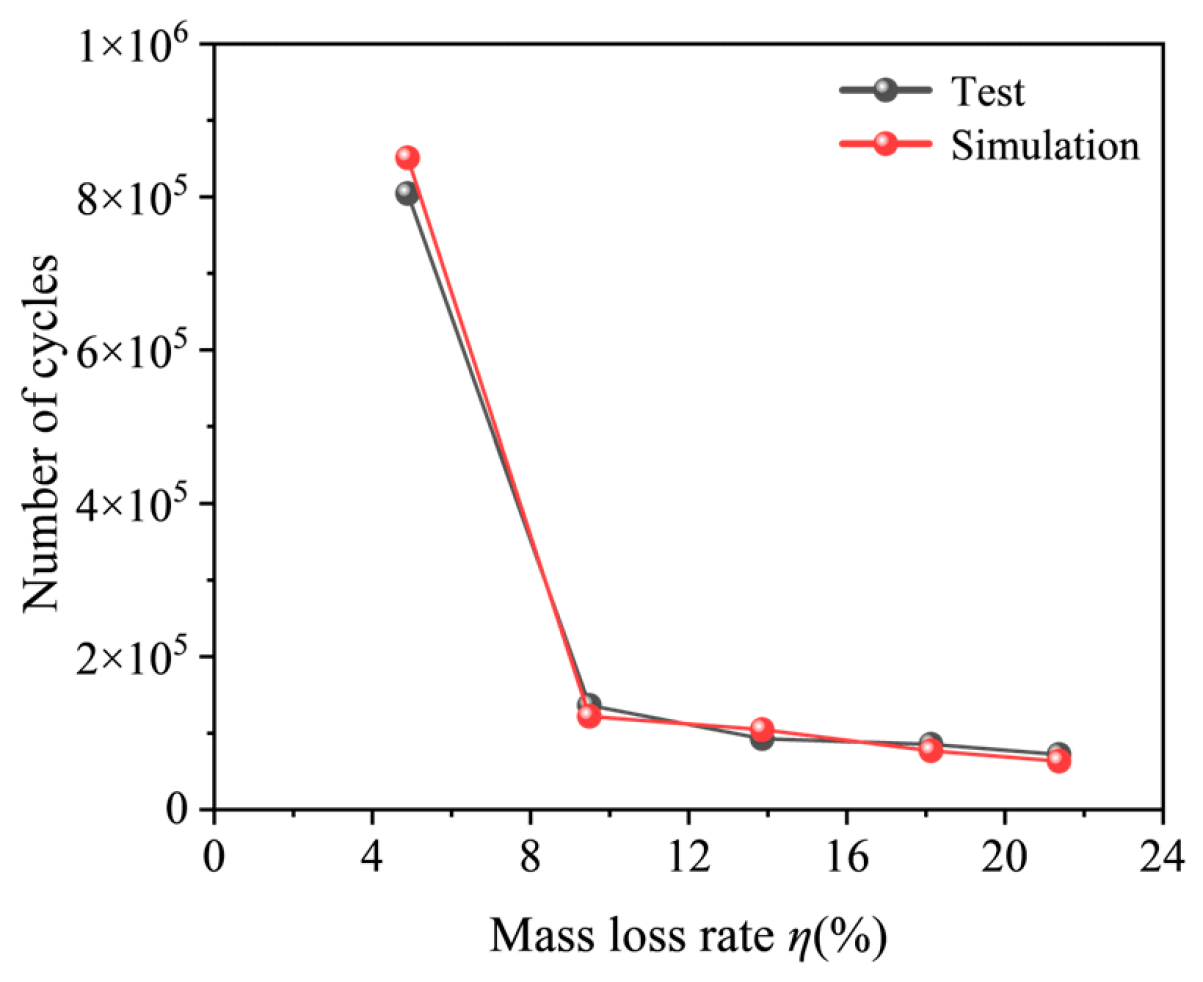
Comparison of simulated and experimental fatigue life of steel wires.

**Figure 18:**
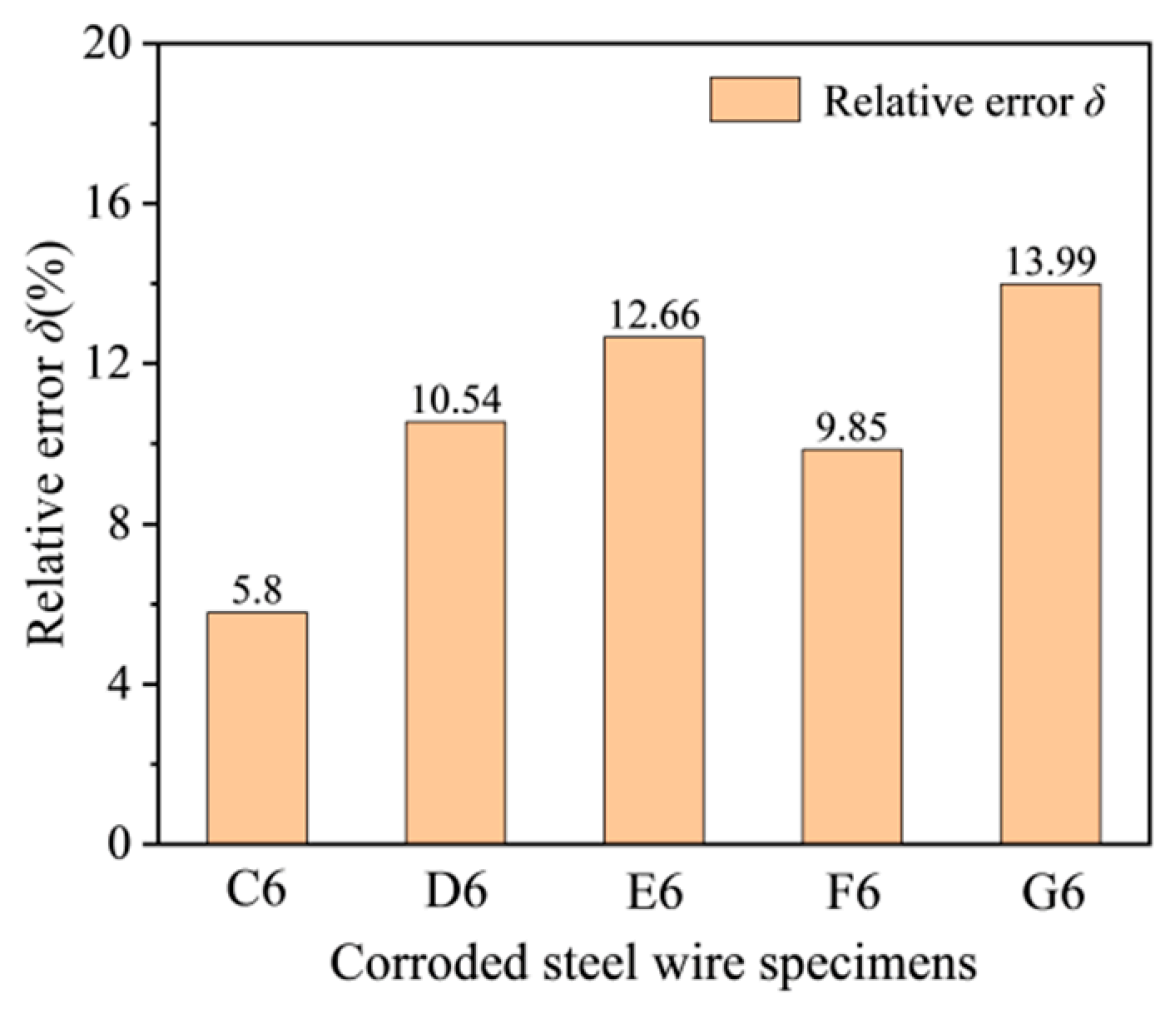
Relative error *δ* of simulated and experimental fatigue life.

Figure 17 compares the simulated fatigue life *N_sim_* with the experimentally measured fatigue life *N_f_* of steel wires under different *η*, while Figure 18 illustrates the corresponding relative error *δ*. It can be observed that the numerically simulated fatigue life is in good agreement with the experimental results, with the relative error δ controlled within 15%. This demonstrates that the 3D scan-reconstructed actual corrosion morphology model can effectively predict the fatigue life of corroded steel wires, validating the reliability and effectiveness of the proposed method. This approach can serve as a methodological reference for the fatigue durability assessment of bridge cables in practical engineering applications.

## 5. Conclusions

- Corrosion significantly degrades the mechanical properties of high-strength galvanized steel wires, with different performance indicators showing varying degrees of sensitivity to corrosion. Although the elastic modulus shows a marginal decrease, it remains relatively insensitive to corrosion. In contrast, both the yield strength and tensile strength decrease with an initially slow decline followed by a more rapid drop with increasing *η*, with the tensile strength undergoing more pronounced degradation. The ductility of the steel wires is highly corrosion-sensitive and deteriorates rapidly even at low corrosion levels. At *η* of 21.54%, the percentage elongation after fracture reduction reaches up to 73.2%, manifesting a significant embrittlement tendency.
- The fatigue life of high-strength galvanized steel wires shows a pronounced exponential decay trend with increasing *η*. Experimentally, fatigue resistance remained largely uncompromised for *η* ≤ 2.75%. However, as *η* increased to 4.84%, fatigue life declined by more than half; at *η* = 21.54%, the fatigue life plummeted to just 3.2% of that recorded for the uncorroded condition.
- Pre-corrosion does not change the essential properties of steel wire materials. Corrosion significantly reduces the fatigue life of high-strength galvanized steel wire, which results from the combined effects of geometric morphology deterioration and mechanical loading effects induced by corrosion.
- Fatigue life predictions derived from steel wire models with actual corroded surfaces reconstructed via 3D scanning-based reverse engineering, combined with Abaqus/fe-safe simulations, are in good agreement with the experimental results, with the relative error controlled within 15%. This validates the applicability of the proposed method for the fatigue durability assessment of bridge cables in practical engineering.

## Figures and Tables

**Table 1:** Measured basic mechanical properties of steel wires.

Number	*E* (GPa)	*σ_y_* (MPa)	*σ_u_* (MPa)	*A* (%)
A1	205.2	1615	1810	5.4
A2	206.2	1627	1819	5.8
A3	206.1	1620	1813	5.7
Mean	205.8	1621	1814	5.6

Note: *E* = Elastic Modulus; *σ_y_* = Yield Strength; *σ_u_* = Tensile Strength; *A* = Percentage Elongation after Fracture.

**Table 2:** Mass loss rate *η* statistics.

Number	*η* (%)	Mean	Number	*η* (%)	Mean	Number	*η* (%)	Mean
B1	2.76	2.75	D1	9.35	9.36	F1	17.84	17.82
B2	2.73		D2	9.32		F2	17.76	
B3	2.75		D3	9.22		F3	17.61	
B4	2.73		D4	9.35		F4	17.53	
B5	2.79		D5	9.41		F5	18.02	
B6	2.76		D6	9.49		F6	18.13	
C1	4.91	4.84	E1	13.52	13.64	G1	22.01	21.54
C2	4.87		E2	13.75		G2	21.71	
C3	4.86		E3	13.31		G3	21.02	
C4	4.78		E4	13.72		G4	21.96	
C5	4.73		E5	13.69		G5	21.19	
C6	4.89		E6	13.83		G6	21.37	

**Table 3:** *S_min_* and *S_avg_* of steel wires under different *η*.

Number	A6	B6	C6	D6	E6	F6	G6
*η* (%)	0	2.76	4.89	9.49	13.83	18.13	21.37
*S_min_* (mm^2^)	38.485	37.502	36.098	33.386	31.337	30.162	27.474
*S_avg_* (mm^2^)	38.485	37.776	36.413	34.535	33.092	31.564	30.545

Note: *S_min_* = Minimum Cross-Section area, *S_avg_* = Average cross-sectional area.

**Table 4:** Fatigue test results of corroded steel wires.

Number	*η*	*N_f_*	Mean	Number	*η*	*N_f_*	Mean
A4	0	>2,000,000		E4	13.72	86,137	
A5	0	>2,000,000	>2,000,000	E5	13.69	107,788	95,624
A6	0	>2,000,000		E6	13.86	92,948	
B4	2.73	>2,000,000		F4	17.53	77,754	
B5	2.79	>2,000,000	>2,000,000	F5	18.02	75,893	79,718
B6	2.76	>2,000,000		F6	18.13	85,509	
C4	4.78	862,894		G4	21.96	52,489	
C5	4.73	1,319,852	995,731	G5	21.19	63,406	63,304
C6	4.89	804,446		G6	21.37	74,019	
D4	9.35	148,137					
D5	9.41	131,228	138,543				
D6	9.49	136,265					

## Data Availability

The original contributions presented in this study are included in the article. Further inquiries can be directed to the corresponding author.
